# Volumetric modulated arc therapy (VMAT) comparison to 3D‐conformal technique in lung stereotactic ablative radiotherapy (SABR)

**DOI:** 10.1002/jmrs.634

**Published:** 2022-11-24

**Authors:** Frances Mark, Anoud Alnsour, Scott N. Penfold, Adrian Esterman, Robert Keys, Hien Le

**Affiliations:** ^1^ Department of Radiation Oncology Royal Adelaide Hospital Adelaide South Australia Australia; ^2^ Oncology Department Plymouth Hospitals Trust Plymouth UK; ^3^ King Hussein Cancer Center –KHCC Amman Jordan; ^4^ Australian Bragg Centre for Proton Therapy and Research Adelaide South Australia Australia; ^5^ Department of Physics University of Adelaide Adelaide South Australia Australia; ^6^ SAHMRI Adelaide South Australia Australia; ^7^ Clinical and Health Sciences University of South Australia Adelaide South Australia Australia

**Keywords:** Non‐small cell lung cancer, radiation pneumonitis, stereotactic ablative radiotherapy, V5, volumetric modulated arc therapy

## Abstract

**Introduction:**

Stereotactic ablative radiotherapy (SABR) can be a curative option for non‐small cell lung cancer (NSCLC) and oligometastatic lung disease. Volumetric modulated arc therapy (VMAT) has offered further advancements in terms of radiation dose shaping without compromising treatment times however there is potential for greater low‐dose exposure to the lung. This study was to assess whether VMAT lung SABR would result in any increase to the dosimetry parameters compared with three‐dimensional conformal radiotherapy (3D‐CRT) that could confer increased risk of radiation pneumonitis.

**Methods:**

A total of 53 and 30 3D‐CRT treatment plans of patients treated with 48 Gy in 4 fractions were compared.

**Results:**

No statistically significant difference in planning target volumes between the VMAT 29.9 cc (range 12.4–58.5 cc) and 3D‐CRT 31.2 cc (range 12.3–58.3 cc) *P* = 0.79. The mean of total lung V5, ipsilateral lung V5 and contralateral lung V5 all showed a trend of being smaller in the VMAT treatment group‐ 14% versus 15.8%, 25.6% versus 30.4% and 1.6% versus 2.2%, respectively, but all were not statistically significant differences. Mean of the mean lung dose MLD, again showed a trend of being lower in the VMAT treatments but was also non‐significant, 2.6 Gy versus 3.0 Gy, *P* = 1.0. Mean V20 was the same in both cohorts, 3.3%.

**Conclusions:**

The dosimetry for 3D‐CRT and VMAT plans were not significantly different including V5, and therefore we conclude that VMAT treatment is unlikely to be associated with an increased risk of radiation pneumonitis.

## Graphical abstract

This paper evaluates the dosimetry of volumetric arc therapy in comparison to 3D‐Conformal technique in delivery of lung stereotactic ablative radiotherapy. No significant dosimetric differences were found between the two techniqiues that could potentially lead to an increased risk of radiation pneumonitis.

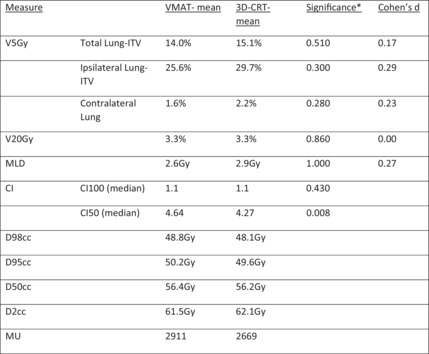

## Introduction

Stereotactic ablative radiotherapy (SABR) can be a precise technique used to deliver an extremely high biological dose of radiation to smaller tumours given in a hypofractionated schedule. It is recognised as standard of care for patients with inoperable stage I non‐small cell lung cancer (NSCLC) based on prospective phase II/III trials that demonstrate a high local control rate of approximately 90%.^1^, ^2^ In Australia, SABR for NSCLC is commonly delivered in 3–8 fractions using biological effective doses in excess of 100 Gy.^3^ SABR has also been recognised as a curative treatment option for oligometastatic disease to the lung.^4^, ^5^

Over time, the technique used to deliver SABR has evolved with advancing linear accelerator technology. Initially, three‐dimensional conformal radiotherapy (3D‐CRT) was utilised, allowing high doses to be delivered to the tumour target whilst relatively avoiding normal structures by modifying static fields. Intensity‐modulated radiotherapy (IMRT) using fixed field beam angles further improved conformity of treatment, but at the expense of longer treatment time for delivery with increased monitor units required. An advanced form of IMRT, volumetric modulated arc therapy (VMAT) has offered further advantages in terms of radiation dose shaping without compromising treatment times, leading to improved patient comfort and accuracy of treatment position and therefore reducing the likelihood of intrafraction tumour displacement.^6^

With coplanar VMAT there is potential for a larger volume of lung to receive low‐dose radiation exposure, for example the volume receiving up to 5 Gy (V5) or volume receiving up to 10 Gy (V10). The clinical significance of this low‐dose exposure remains uncertain. Radiation pneumonitis (RP) is a major dose‐limiting toxicity in the treatment of lung tumours, where dose‐volume values may be a predictor of RP.^7^ In conventional fractionated radiotherapy, the Quantitative Analysis of Normal Tissue Effects in the Clinic (QUANTEC) recommends a V20 of ≤30% to 35% and a mean lung dose (MLD) of ≤20 Gy to 23 Gy to reduce the risk of developing RP to less than 20%.^7^ Other studies have found V5, along with other dosimetry parameters, to be a potential predictor of RP in conventionally fractionated radiotherapy^8^, ^9^, ^10^, ^11^, ^12^, ^13^, ^14^ and SABR.^15^, ^16^, ^17^, ^18^, ^19^, ^20^

In this study, a retrospective dosimetric comparison of 3D‐CRT and VMAT lung SABR treatment plans was performed. The plans were generated and delivered at the Royal Adelaide Hospital (RAH), South Australia, between 2014 and 2020. The purpose of the study was to compare dose‐volume metrics between the two planning approaches, with a particular focus on those related to RP such as the volume receiving up to 5 Gy (V5).

## Methods and Materials

This study was conducted under Human Research Ethics Committee (HREC) approval, Central Adelaide Local Health Network (CALHN) Ethics committee, reference number 13751. Treatment plans created at the RAH between February 2014 (VMAT started Aug 2018) and October 2020 were included in the study. Patients with inoperable stage I‐II NSCLC (biopsy proven or radiological diagnosis) and oligometastatic lung disease regardless of primary site were included. Only those patients prescribed the departmental standard fractionation of 48 Gy in 4 fractions to a single site were considered in the study.

Treatment plans utilising isotropic 0.5 cm expansion margin from internal target volume (ITV) to planning target volume (PTV) were included. Patients with previous lung resections were excluded. Fixed field IMRT was used to deliver lung SABR treatments prior to the adoption of VMAT; however, these plans were excluded from analysis (10 patients).

Initially, 62 VMAT plans and 46 3D‐CRT plans meeting the above criteria were retrieved. It was observed that the PTVs in the 3D‐CRT cohort were significantly larger than the PTVs in the VMAT cohort. As the volume of the PTV directly impacts the dose‐volume histogram (DVH) metrics of particular interest in this study (V5 and V10), the inclusion criteria were further restricted to ensure comparable patient cohorts were being considered. Analysis showed that the VMAT and 3D‐CRT cohorts were well matched if PTV volumes were restricted to 10–60 cc. Removing PTV volumes outside this range from both cohorts resulted in median PTVs of 29.9 cc and 31.2 cc for the VMAT and 3D‐CRT cohorts, respectively (mean PTV 31.2 cc VMAT and 32.1 cc 3D‐CRT). After these exclusions the difference between the median PTVs was not statistically significant and these plans were used for further analysis. Therefore, the VMAT cohort consisted of 53 treatment plans and the 3D‐CRT cohort 30 treatment plans.

All patients were imaged with four‐dimensional computed tomography (4DCT). The gross tumour volume (GTV) was contoured on the maximum intensity projection (MIP) image set and the 0% and 50% respiratory phases with the aid of PET fusion. An internal target volume (ITV) was created on the 4D average data set (AVG) as the union of the GTV across these three data sets. An isotropic expansion of 0.5 cm was added to the ITV to create the PTV. Dose calculation was performed on the AVG data set.

The Pinnacle treatment planning system (TPS) was used for all plans produced by different radiotherapy planners over the 6 years used for this study.^21^ The plans were reviewed weekly by the SABR multidisciplinary team to ensure consistent quality. All treatments were planned with a 6 MV X‐ray beam. 56% of patients in the 3D‐CRT cohort utilised a flattening filter‐free (FFF) beam, compared to 100% in the VMAT cohort. Commissioning of FFF occurred after the initial release of SABR with 3D‐CRT. Commissioning of VMAT for lung SABR occurred in 2018, 4 years after commissioning of 3D‐CRT for lung SABR.

3D‐CRT plans were created with between 8 and 12 non‐opposing beams, typically including 2 non‐coplanar beams. No beams overlapped at the skin surface and where possible beams entering through the contralateral lung were avoided. The collimator angle was varied for each beam.

VMAT plans typically consisted of 2 co‐planar partial arcs. To reduce dose delivery uncertainties due to interplay, attempts were made to limit MLC modulation such that the plan delivers less than 300 MU/Gy, where the prescription dose per fraction is used for normalisation.

The treatment planning guidelines and objectives followed the CHISEL trial organ at risk constraints for 48 Gy in 4 fractions including lung V20 < 15% and mean lung dose (MLD) <20 Gy.^1^ The dose of 48 Gy was typically prescribed to the 80% isodose line delivering a BED of >100 Gy.

Patient and treatment data were collected from the oncology information system, electronic records and TPS. Patient demographics included age, sex and performance status (PS); tumour information included side, lobe, histopathology and lesion diameter. The treatment delivery and planning details including PTV (cc), PTV mean dose, total MLD, planning target volume coverage metrics of D2cc (Gy), D50cc (Gy), D95cc (Gy) and D98cc (Gy), monitor units delivered, contralateral and ipsilateral lung V5 Gy (%) and total lung V20 Gy (%) and V5 Gy (%) (both minus ITV). Conformity index (CI) 100 and CI50 were calculated from the above data as per ICRU 91,^22^ which were used as per the TROG CHISEL study.^1^, ^22^ The formulae used were,

$${\mathrm{CI}}_{\mathrm{iso}}=\frac{V_{\mathrm{iso}}}{V_{\mathrm{PTV}}},$$

where *V*_*iso*_ is the volume encapsulated by the respective isodose line and *V*_*PTV*_ is the volume of the *PTV*.

### Statistical procedures

This is an exploratory study, and as such, no formal power calculation was undertaken. Patient characteristics between the two groups were compared using one‐sided chi‐squared tests. Dosimetric differences were examined with two‐sided independent sample t‐tests with unequal variance to account for different sample sizes. Differences between medians were compared using median tests. *P* < 0.05 was considered statistically significant. Stata 16 was used for all statistical analyses.

## Results

### Patient and tumour characteristics

The patient and tumour characteristics of the 53 VMAT and 30 3D‐CRT lung SABR cohorts were compared as shown in Table 1. The median age of the patients for VMAT and 3D‐CRT were 75 yrs (range 22–92 yrs) and 71 yrs (range 48–91 yrs), respectively. The difference was not statistically significant using a median test (*P* = 0.056). The majority of patients were PS 0–1 (>80% in both cohorts). There were more patients with a radiological diagnosis of lung cancer/metastasis in the VMAT arm. Sex, PS, laterality, lobe and histopathology were not statistically significantly different between the VMAT and 3D‐CRT treatment groups (table 1). Although there were more male patients in 3D‐CRT group, did not confer any difference in total lung volume (*P* = 0.79).

**Table 1:** Patient and tumour characteristics of the 53 VMAT and 30 3D‐CRT SABR treatments.

Descriptor	VMAT‐ *N* (%)	3D‐CRT‐ *N* (%)	Significance*
Sex			
Male	20 (38)	17 (57)	0.098
Female	33 (62)	13 (43)	
PS			
0–1	45 (85)	25 (84)	0.404
2–3	8 (15)	4 (13)	
Unknown		1 (3)	
Laterality			
Left	23 (43)	11 (37)	0.552
Right	30 (57)	19 (63)	
Lobe			
LUL	15 (28)	6 (20)	0.520
LML	1 (2)	0	
LLL	7 (13)	4 (13)	
RLL	13 (25)	5 (17)	
RUL	17 (32)	15 (50)	
Histology			
Adenocarcinoma	17 (32)	14 (47)	0.370
Squamous cell	5 (9)	5 (17)	
Large cell	1 (2)	1 (3)	
Metastatic	10 (19)	3 (10)	
Radiological	20 (38)	7 (23)	

* One‐sided chi‐squared test, PS, performance status.

Abbreviations:3D‐CRT, three‐dimensional conformal radiotherapy; LLL, left lower lobe; LML, left middle lode; LUL, left upper lobe; RLL, right lower lobe; RUL, right upper lobe; VMAT, volumetric arc therapy.

### Treatment

All patients in this retrospective study received 48 Gy in 4 fractions. The VMAT treatments were all delivered with partial arcs; 51 (96%) plans with 2 arcs, 2 plans with 3 and 4 arcs each. 3 of the 53 VMAT plans included one non‐coplanar arc. The 3D‐CRT plans were all non‐coplanar, 27 plans using 10 beams (60%), 7(16%) with 8–9 beams and 11(24%) with 11–12 beams. All 3DCRT plans included at least 1 beam with a non‐zero couch angle.

There was no statistically significant difference between the median maximum diameter of tumour GTV between the cohorts; VMAT 15 mm (range 7–33 mm) and 3D‐CRT 18 mm (range 9–41 mm) *P* = 0.16. Nor was there a statistically significant difference in median planning target volumes between the VMAT 29.9 cc (range 12.4–58.5 cc) and 3D‐CRT 31.2 cc (range 12.3–58.3 cc) *P* = 0.79. Dosimetry differences between the two treatment techniques are shown in Table 2.

**Table 2:** Dosimetry of patients that received VMAT (*n* = 53) and 3DCRT (*n* = 30) planning techniques.

Measure		VMAT‐ mean	3D‐CRT‐ mean	Significance*	Cohen's d
V5 Gy	Total Lung‐ITV	14.0%	15.1%	0.510	0.17
	Ipsilateral Lung‐ITV	25.6%	29.7%	0.300	0.29
	Contralateral Lung	1.6%	2.2%	0.280	0.23
V20 Gy		3.3%	3.3%	0.860	0.00
MLD		2.6 Gy	2.9 Gy	1.000	0.27
CI	CI100 (median)	1.1	1.1	0.430	
	CI50 (median)	4.64	4.27	0.008	
D98cc		48.8 Gy	48.1 Gy		
D95cc		50.2 Gy	49.6 Gy		
D50cc		56.4 Gy	56.2 Gy		
D2cc		61.5 Gy	62.1 Gy		
MU		2911	2669		

* Based on two‐sided independent samples t‐tests for mean comparisons, and two‐sided Median test for median comparisons.

Abbreviations: CI, conformity index; ITV, internal target volume; MLD, mean lung dose; MU, monitor units.

D98cc/D95cc/D50cc/D2cc Planning Target Volume coverage metrics.

The mean of the total lung V5, ipsilateral lung V5 and contralateral lung V5 all showed a trend of being smaller in the VMAT treatment group‐ 14% versus 15.8%, 25.6% versus 30.4% and 1.6% versus 2.2%, respectively, but all were not statistically significant differences. Mean of the MLD, again showed a trend of being lower in the VMAT treatments but was also non‐significant, 2.6 Gy versus 3.0 Gy, *P* = 1.0. Mean V20 was the same in both cohorts, 3.3%.

The median CI100 was 1.1 in both treatment cohorts, but for the median VMAT CI50 was significantly higher at 4.64 versus 4.27 for 3D‐CRT (*P* = 0.008). The monitor unit (MU) count was higher in the VMAT cohort, but there was no statistically significant difference.

## Discussion

### Delivery of SABR plans

**Figure 1:**
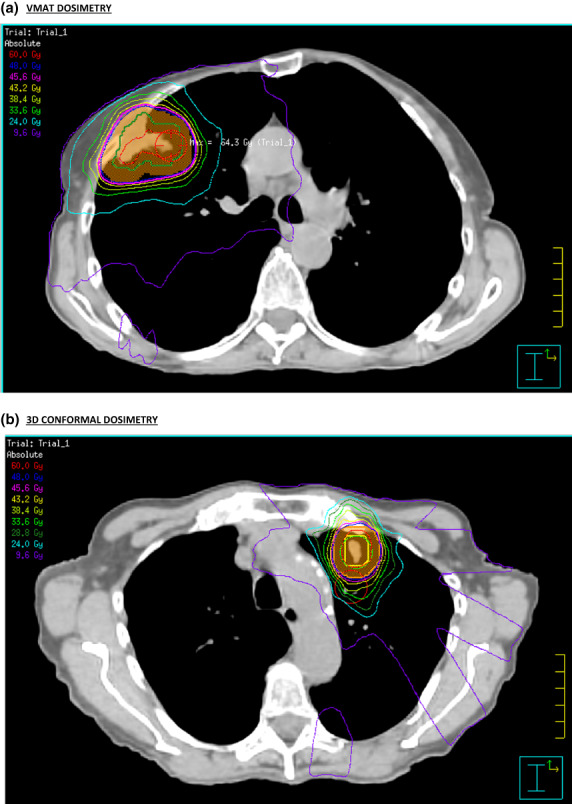
(A) and (B) VMAT and 3D‐CRT dosimetry.

SABR for early‐stage primary NSCLC and oligometastatic disease to the lung can offer high local control rates with minimal toxicity.^1^, ^2^ The advantage of SABR is in delivering ablative radiotherapy dose to the tumour target whilst relatively avoiding normal structures with good conformity to the PTV and steep dose drop‐off outside the PTV. The conformity index, CI100 was non‐significantly different between the VMAT and 3D‐CRT plans in our study. The CI50 for 3D‐CRT (mean 4.27) was significantly lower than VMAT (mean 4.64) in our study. A steeper dose‐gradient can potentially be created in non‐coplanar 3D‐CRT plans, compared with coplanar VMAT (93% coplanar in our study), dosimetry differences in VMAT and 3D‐CRT plans are shown in Figure 1.

Excellent PTV conformity can be achieved with VMAT plans. Fitzgerald et al compared 3D‐CRT versus IMRT versus VMAT lung non‐coplanar SABR plans for each of 20 patients in contrast to comparing plans for different patients as in our study.^23^ Fitzgerald et al found VMAT to create the greatest PTV conformity, this has also been seen in other planning studies.^24^, ^25^, ^26^ Navarria et al reviewed 86 3D‐CRT and 46 coplanar VMAT plans delivering lung SABR and found no difference in conformity, although it was not clear which CI calculation was used.^25^ Further information on dose conformity levels can be evaluated with by giving tolerance and minor deviation values for R100% and R50% metrics as per the ROSEL trial, but the data collected for this study was prior to this progression.^27^

### Dosimetry metrics

Dosemetrics to quantify the dose to normal lung as an organ at risk, conventional fractionation have been well‐studied, and V20 of ≤30% to 35% and MLD of ≤20 to 23 Gy are often used to predict RP <20%.^7^ A recent systematic review of lung SABR reported most studies note safe treatment with MLD of combined lungs ≤8 Gy in 3–5 fractions and V20 < 10–15% 0.24. Our VMAT study results of median MLD of 2.6 Gy and median V20 of 3.3% are well below these constraints and those of previous SABR studies (shown in table 3).

**Table 3:** SABR dosimetry parameters for potential prediction of radiation pneumonitis (RP).

Study	Patient number	Median PTV (cc)	SABR dose/fractionation	Delivery method	Pneumonitis grade assessed	Low dose RP predictive dosimetric parameters	Threshold for low dose RP	*P* value for low dose RP	Other RP Predictive dosimetric parameter	Threshold for other dosimetryRP
Ricardi^28^ (2009)	60	NA	45 Gy/3^#^, 26 Gy/1^#^	3D‐CRT	≥3	Not investigated	‐	‐	MLD	12 Gy
Guckenberger^15^ (2010)	59	33	26 Gy/1^#^, 37.5/3^#^	3D‐CRT	≥2	V2.5	NA	NA	MLD V2.5–50	12.5 Gy NA
Ong^16^ (2010)	18	137	55 Gy/5^#^, 60 Gy/8^#^	VMAT	≥3	Total V5 contralateral V5 ipsilateral V5	37% 26% NA	<0.0001 <0.0001 0.004	MLD	NA
Takeda^17^ (2011)	128	NA	50 Gy/5^#^ (50 Gy/10)^#^, 40 Gy/5^#^ (60 Gy/5)^#^	DCAT^†^	0–1 vs 2	V5‐15	NA	<0.0001	MLD V5‐50	NA NA
Barringer^29^ (2012)	251	48.3	24–66 Gy/3–5^#^	3D‐CRT	≥2	‐	‐	0.02 0.03	Total MLD V20	4 Gy 4%
Matsuo^30^ (2012)	74	32.5	48 Gy/4^#^	3D‐CRT	≥2	‐	‐	‐	V20 V25 PTV	5.8% 4.2% 37.7 cc
Chang^18^ (2012)	130	73.2	50 Gy/4^#^	3D‐CRT	2–3	V5 V10 V15	20.2% 14.3% 11%	<0.001 <0.001 0.003	MLD V40 V30	5.1 Gy 3.1% 5%
Baker^19^ (2013)	240	37.6	40–60 Gy/4–8^#^	NA	≥2	V5 V13	NA NA	0.0186 0.0438	‐	‐
Nakamura^20^ (2016)	56	23.8	48–56 Gy	Cyberknife	≥2	V5 V10 V15	27% 16.3% 10.6%	0.008 0.002 0.001	V5‐50 MLD PTV GTV	‐ 5.1 Gy 56.7 cc 13.4 cc
Kim^31^ (2017)	59	14.4	45–60 Gy/3–4^#^	VMAT/Cyberknife/Tomo^‡^	≥2	‐	‐	‐	ITV PTV	4.2 cc 14.4 cc
Saha^32^ (2021)	1266	30.3	50–60 Gy/3–8^#^	3D‐CRT	≥2	V12.5	9.5%	0.000	V20 MLD PTV	4.6% 3.7 Gy 27.15 cc

^#^ Fractions.

^†^ DCAT, Dynamic Conformal Arc Therapy.

^‡^ Tomo, Tomotherapy.

NA‐ Not available.

In the literature, there is debate regarding a potential increase of low dose radiation with VMAT delivery of lung radiotherapy. Ong et al compared 3D‐CRT and VMAT for each of 18 lung SABR patients and found a small increase in V5 to contralateral lung with VMAT to large volume lung tumours; 1.2% versus 4.4% *P* = 0.011.^16^ Chan et al's dosimetric conventional fractionation (stage III NSCLC) study of 15 patient plans found the V5, V10 and V15 were significantly higher in VMAT than 3D‐CRT.^26^

However, several smaller PTV lung SABR studies have found a reduction in low dose radiotherapy volumes with VMAT, including Navarria et al, Zhang et al and McGrath et al.^6^, ^25^, ^33^ Zhang et al compared non‐coplanar 3D‐CRT, coplanar and non‐coplanar VMAT, and FFF VMAT plans for each of 15 patients.^33^ Total lung V5 was lowest in the FFF VMAT plans. McGrath et al found V12.5, V10 and V5 to be significantly lower in VMAT versus 3D‐CRT plans created for each of 21 patients.^6^ These findings align with our own study, in finding the VMAT delivery of lung SABR does not increase the low dose radiation to the lung. We found no statistically significant difference in median total V5, ipsilateral V5, contralateral V5 between VMAT versus 3D‐CRT delivery of Lung SABR.

### Radiation pneumonitis risk with low dose radiation

Radiation pneumonitis is the main dose‐limiting toxicity for lung radiation, reported rates after lung SABR range from 9% to 28%.^34^ The hypothesis that low, as well as higher radiation doses, are predictive for the development of RP is supported by several studies. Gopal et al used^35^ pulmonary diffusion capacity (DLCO) to estimate for loss of lung function and investigated relative to dosimetric parameters. Grade 2 or higher RP was associated with a DLCO loss of >30% (*P* = 0.003) and this loss of function was greatest for V13 (95%, CI 11–15 Gy). Palma et al and Kyas et al both used CT to assess density changes as an objective measure of lung damage post‐SABR, which has been found to correlate with histological findings of inflammation.^36^, ^37^ Density increases were apparent in areas from above 6 Gy.

Guckenberger et al investigated 3D‐CRT lung SABR, concluding that ipsilateral lung V2.5 best correlated with development of RP (using a range of 2.5–50 Gy).^15^ Ong et al reported contralateral lung V5 to be the most significant parameter to predict RP ≥ G2 (*P* < 0.0001) when using VMAT SABR for relatively large lung tumours (median volume 137 cc).^16^ From this study V5 predictive of RP with SABR was reported as 26% for contralateral lung and 37% for ipsilateral lung using VMAT. Takeda et al reported the V5 of the lung SABR plans was significantly higher in patients with Grade 2 RP versus Grade 0–1 (23.3% vs 17.7% *P* < 0.0001).^17^

### Future implications

As the concept of aggressive management of oligometastatic disease has been supported by recent data (Palma et al), the issue of thoracic re‐irradiation is an important consideration.^38^ Ren et al investigated dosimetry in the prediction of RP for re‐irradiation with IMRT/3D‐CRT delivery of convention fractionated radiotherapy or SABR.^9^ They showed composite V5 and the overlap of V5/re‐irradiated V5 volume both to be predictors of RP. Medium overlap of V5 volumes (0.4–0.8) were significantly associated to predict RP; hypothesising the lung had been primed by the <V5 low dose bath. We may see the incidence of RP rise as retreatment lung SABR increases, as well as with the potential increased use of SABR with chemotherapy and/or immunotherapy.

### Limitations

This was a retrospective, single‐institution study. Although this is the largest study reporting dosimetric differences between 3D‐CRT and VMAT lung SABR, the sample size is still relatively small and therefore the statistical analysis less reliable. The SABR plans being produced be different planners over the years of the study and the team gaining greater experience over time is a confounding factor of the study. This was a dosimetric study only and was not directly correlated with clinical outcomes such as rate of RP.

## Conclusion

The aim of this study was to assess whether VMAT lung SABR would result in any increase to the dosimetry parameters compared with 3D‐CRT that could confer increased risk of radiation pneumonitis. From this retrospective analysis of comparable PTV treatments, the dosimetry for 3D‐CRT and VMAT plans were not significantly different including V5, and therefore we conclude that VMAT treatment is unlikely to be associated with an increased risk of RP.

Lower dose volumes may increasingly predict RP in lung SABR planning for larger PTVs, retreatment or when combining with chemotherapy/immunotherapy. The most accurate low‐dose volume threshold percentages to predict radiation pneumonitis post‐SABR are yet to be defined.

## Acknowledgements

All of the Radiation Oncology team at the Royal Adelaide Hospital.

## Conflict of interest

The authors declare no conflict of interest.
